# Radiofrequency Ablation of an Atrial Tachycardia Emanating From the Non-coronary Aortic Cusp Guided by an Electroanatomic Navigation System

**Published:** 2010-02-01

**Authors:** Agustin Bortone, Eric Maupas

**Affiliations:** Departement de Rythmologie. Hopital Prive Les Franciscaines. Unite de Cardiologie. 9, Impasse Jean Bouin. Nimes, France.

**Keywords:** Atrial tachycardia, non-coronary aortic cusp, radiofrequency ablation, Carto

## Abstract

We report on an atrial tachycardia (AT), emanating from the non-coronary (NC) aortic cusp, ablated with the aid of an electro-anatomical navigation system. In this setting, the electrocardiographic, electrophysiologic (EP), anatomical, and ablative considerations are discussed.

Although NC aortic cusp focal ATs are an uncommon EP finding, their ablation is effective and safe, especially from an atrio-ventricular (AV) conductive point of view. This origin of AT must be invoked and systematically disclosed when a peri-AV nodal AT origin is suspected, in order to avoid a potentially harmful energy application at the vicinity of the AV conductive tissue.

## Case Presentation

**Figure 1:**
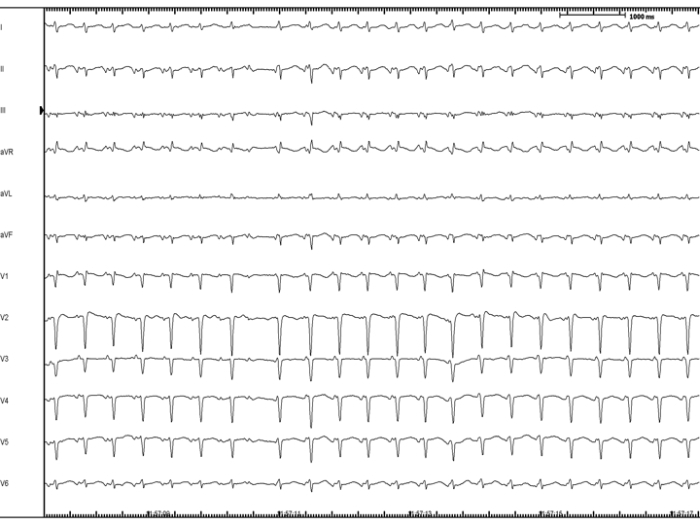
Twelve-lead electrocardiogram recorded during the long RP clinical tachycardia

**Figure 2:**
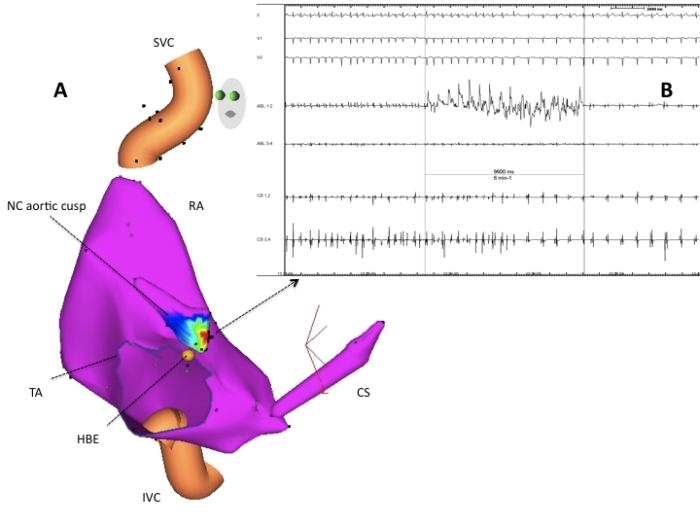
Panel A: Three-dimensional activation map of the right atrium (RA), the coronary sinus (CS) and the non-coronary (NC) aortic cusp constructed, using Carto, during focal atrial tachycardia (AT). The small red color region corresponds to the maximal precocity site, which is localized at the NC aortic cusp. Panel B:  Termination of the focal AT while applying radiofrequency energy at the region presenting the maximal precocity (red region). Are shown surface II, V1 and V3 leads, distal and proximal electrodes from the ablation catheter (ABL 1-2 and ABL 3-4) and distal and proximal electrodes from the quadripolar catheter inserted within the coronary sinus (CS 1-2 and CS 3-4). TA = tricuspid annulus, SVC = superior vena cava, IVC = inferior vena cava, HBE = His bundle.

**Figure 3:**
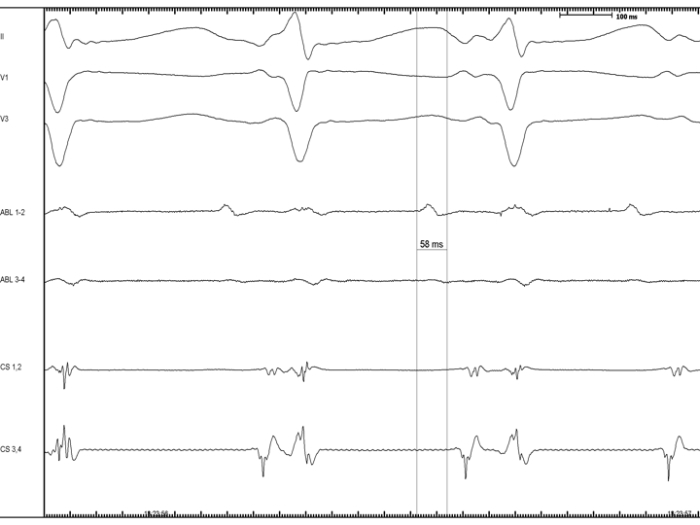
Intra-cardiac electrograms during focal AT while mapping in the NC aortic cusp. The mapping catheter exhibits an atrial electrogram that precedes the onset of the P wave by 58 ms. Are shown surface II, V1 and V3 leads, distal and proximal electrodes from the ablation catheter (ABL 1-2 and ABL 3-4) and distal and proximal electrodes from the quadripolar catheter inserted within the coronary sinus (CS 1-2 and CS 3-4). Please note that the focal AT cycle length is variable.

**Figure 4:**
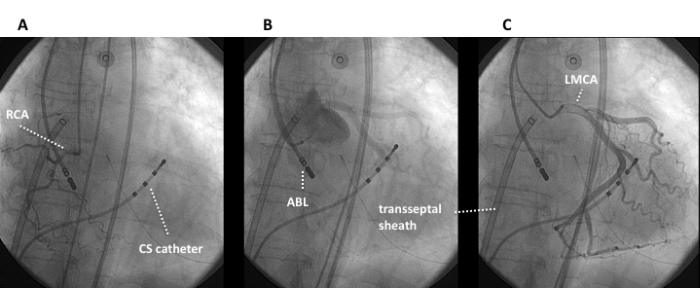
Fluoroscopic images taken while the ablation catheter recorded the earliest atrial electrogram (corresponding to the red region shown in Figure 2-panel A and as shown in Figure 3). Panels A: contrast injection of the right coronary artery (RCA). Panel B:  Non-selective injection of the sinuses of Valsalva. Panel C: contrast injection of the left mean coronary artery (LMCA). All images demonstrate a safe distance between the coronary arteries and the ablation catheter (ABL).

A 37 year-old woman, without any structural heart disease, was referred to our institution for management of symptomatic supra-ventricular tachycardia. Three anti-arrhythmic drugs had failed before the patient was considered for catheter ablation. Baseline 12-lead electrocardiogram (ECG) and echocardiogram were both normal. After written informed consent was obtained, an EP study was undertaken. A narrow complex tachycardia was easily induced by simple catheter manipulation (Figure 1). Upon cessation of ventricular pacing with capture at 20 ms faster than the tachycardia cycle length a V-A-A-V response was observed, which assessed the diagnosis of AT. Right atrium (RA) and coronary sinus (CS) activation maps were constructed using Carto™ (Biosense Webster, Diamond Bar, CA.). The earliest activation region was located at the initial portion of the bundle of His. Afterwards, left atrium (LA) mapping was performed after transseptal access was obtained. No activation maps of the LA and the pulmonary veins were built because mapping was poor in terms of precocity. After retrograde aortic approach was performed, a NC aortic cusp activation map was created and exhibited the maximal precocity (Figure 2-panel A and Figure 3). A 10 seconds RF application at the NC aortic cusp site (25 W, 48ºC, 30 cc/min) abolished the AT (Figure 2-panel B) and rendered this one no more inducible with and without isoproterenol administration. Prior to RF application, right and left coronary angiograms demonstrated that the ablation catheter was located at a safe distance with respect to the origin of the coronary arteries (Figure 4). There were no complications. During the one-year follow-up period the patient had an ECG Holter monitoring at months 1, 3, 6 and 12, and was invited to come to the ablation center in case of symptoms suggestive of AT recurrence. During all that period, the patient has been arrhythmia free without any anti-arrhythmic medication.

## Discussion

It has been shown that the right and left aortic coronary cusps can give rise to ventricular tachycardia [1,2] while the NC aortic cusp can be a source of AT [2-6]. Although ATs emerging from the NC aortic cusp are uncommon; the safety of their ablation in terms of AV conduction disturbances, aortic leaflets integrity and embolic events has been assessed [2-4].

On the other hand, the fact that the interatrial septum and the NC aortic cusp are immediately adjacent [5], led some investigators to postulate that some aborted AT ablations presumed to emerge from the peri-AV nodal region could have been in fact related to a NC aortic cusp origin [2]. Accordingly, it seems reasonable to map the NC aortic cusp after mapping the left aspect of the interatrial septum and the initial portion of the CS when an AT, presumably emerging from the peri-AV nodal region, is diagnosed [2]. Furthermore, RF application at the right aspect of the interatrial septum while targeting peri-AV nodal ATs carries a substantial risk of complete heart block, while RF application to the NC aortic cusp, is less dangerous; unless the RF is applied near the commissure with the right coronary cusp because at that location, the fast-pathway can be injured [5].

When mapping the posterior and left-sided aspects of the NC aortic cusp, large atrial electrograms must be recorded whereas, when the catheter approaches the commissure with the right coronary cusp, His bundle electrograms and large ventricular electrograms appear. Although, atrial NC aortic cusp atrial electrograms have been described as fractionated [5,7] in our case they were not fractionated (Figure 3), suggesting an abnormal automaticity mechanism (as assessed by the impossibility to entrain the AT and its spontaneous cycle length variations) or a supra-valvular myocardium origin. These assertions remain however, speculative.

Typically, NC aortic cusp focal AT 12-lead ECG shows positive P waves in V1, while an anterior tricuspid valve origin or/and a RA appendage origin, both exhibit a negative P wave pattern in V1 [4]. Interestingly, in our case the ECG shows a positive/negative P wave in V1 (Figure 1) which can correspond to an anatomical variation of the AT origin within the space between the NC aortic cusp and the interatrial septum or a variation of the patient's anatomy.

Regarding the energy used for NC cusp ablation, recent publications, either experimental or clinical, have assessed the security of standard RF energy, cooled-tip RF energy and cryothermal ablation [1-8]. In our case, we have chosen irrigated RF energy only because the available catheter in our EP laboratory, at the moment of the procedure, compatible with the Carto™ system was irrigated. However, standard RF ablation and cryothermal ablation may be sufficient.

Concerning electroanatomical navigation systems, although non-indispensable, they can depict comfortably the activation of all the chambers mapped and allow the reproducible repositioning of the ablation catheter at the "spot of interest". These systems have therefore the potential to render easer the ablation procedures. Moreover, merging the computed tomography of the heart with the electro-anatomic reconstruction may add further information, enhance the understanding of the complex anatomic relationship between structures, and increase safety at the level of coronary cusps [9,10].

Finally, the distance between the ablation catheter and the origin of the coronary arteries should be absolutely disclosed by means of a prior coronary angiogram, in order to avoid a serious coronary complication [2].

## Conclusion

Although ATs originating from the NC aortic cusp are uncommon, their ablation is feasible and safe. NC aortic cusp ATs must be invoked and systematically disclosed when a peri-AV nodal AT origin is suspected, in order to avoid a potentially harmful energy application at the vicinity of the AV conductive tissue.
